# A Hierarchical Framework Combining Motion and Feature Information for Infrared-Visible Video Registration

**DOI:** 10.3390/s17020384

**Published:** 2017-02-16

**Authors:** Xinglong Sun, Tingfa Xu, Jizhou Zhang, Xiangmin Li

**Affiliations:** 1 School of Optoelectronics, Image Engineering & Video Technology Lab, Beijing Institute of Technology, Beijing 100081, China; 2120545@bit.edu.cn (X.S.); xiaomianzhou@126.com (J.Z.); li_xiangmin@bit.edu.cn (X.L.); 2 Key Laboratory of Photoelectronic Imaging Technology and System, Ministry of Education of China, Beijing 100081, China

**Keywords:** infrared-visible registration, objective motion vector, normalized location, edge orientation, mismatch elimination

## Abstract

In this paper, we propose a novel hierarchical framework that combines motion and feature information to implement infrared-visible video registration on nearly planar scenes. In contrast to previous approaches, which involve the direct use of feature matching to find the global homography, the framework adds coarse registration based on the motion vectors of targets to estimate scale and rotation prior to matching. In precise registration based on keypoint matching, the scale and rotation are used in re-location to eliminate their impact on targets and keypoints. To strictly match the keypoints, first, we improve the quality of keypoint matching by using normalized location descriptors and descriptors generated by the histogram of edge orientation. Second, we remove most mismatches by counting the matching directions of correspondences. We tested our framework on a public dataset, where our proposed framework outperformed two recently-proposed state-of-the-art global registration methods in almost all tested videos.

## 1. Introduction

With the development of sensors, multi-sensor image fusion has attracted a considerable amount of research interest in recent years. Particular attention has been devoted to infrared-visible sensor fusion [1,2,3], which adequately uses spectrum information in different wavebands of the same scene. It is helpful in strengthening the complementarity of scene information and reducing uncertainty in scenario analysis. This fusion has been extensively applied to human detection [4], visual surveillance, target tracking, face recognition [5] and so on. In many cases of fusion, registration is needed to automatically find the transformation matrix between two images (or videos). Accurate image registration can eliminate the influence of various sensor positions, and can generate more distinct appearances and boundaries of targets following fusion.

However, it is challenging to find correspondences in infrared-visible images for registration because they reflect different phenomena [6]. Infrared images record heat radiations emitted by objects, whereas visible images record reflected light on objects. There is an obvious difference in intensity between infrared and visible pixels, which means that intensity-based methods are rendered useless. Moreover, a visible texture is often missing in the infrared image because it seldom influences heat emitted by an object. In summary, infrared-visible registration continues to be a challenging problem.

To solve the above problem, we propose, in this study, a novel automatic registration framework based on curvature scale space (CSS) keypoints [7] for infrared-visible videos. Most feature-based methods proposed in past work, such as [8,9,10], which directly adopt feature matching, find it difficult to obtain accurate correspondences to find the global homography. Such motion-based methods as [11,12] cannot implement the registration in a complex scenario. In contrast to these, our proposed framework adopts a hierarchical registration method by combining motion and feature information, which includes coarse and precise registration. In coarse registration, we use CSS keypoints to estimate the motion vectors of targets, which are used to calculate scale and rotation for the entire video pair. In precise registration, we first re-locate targets and keypoints according to the results of coarse registration, and then construct a novel matching strategy for infrared and visible keypoints. The correspondences are stored in a new reservoir to find the best global transformation matrix. As in other motion-based registration methods, we suppose that there are moving objects in the observed scene, which are synchronized in the infrared-visible videos. During the video acquisition process, the relative position between the infrared and visible cameras is fixed, and the intersecting fields of view of the two cameras are required.

Our contributions in this paper are summarized as follows:

1. We propose a new registration framework based on CSS keypoints that improves the accuracy of global homography by combining feature and motion information.
2. We propose a simple method to calculate the motion vectors of targets in coarse registration that transforms the scale and rotation estimation into an easy, homologous keypoint-matching problem.
3. We calculate the normalized location (NL) descriptors and the histogram of edge orientation (HOE) descriptors to strictly match keypoints. Moreover, we count the matching directions of correspondences to eliminate mismatches.
4. We use a reservoir where correspondences that are identified as outliers based on the HOE matching metric are randomly replaced. Compared to the reservoirs proposed in [13,14], ours can save a sufficient number of representative matches for registration.

The rest of this paper is organized as follows: Section 2 explores related work in the area, whereas Section 3 introduces our proposed method. Section 4 provides a description of our experiments and their results, and we offer our conclusions in Section 5.

## 2. Related Work

In the domain of image registration, various methods have been studied to reduce computation time [15] and improve the precision of registration [16]. These can be classified into three types: (1) intensity-based methods; (2) feature-based methods; and (3) motion-based methods. We briefly review these methods below.

In intensity-based methods, image region correlation [17] or mutual information [6,18,19,20,21] has been used to find homography. Given two images, the region correlation methods consider the window pair with the largest similarity as a correspondence [17]. These methods have some drawbacks, such as the flatness of the similarity measure in textureless regions and high computational complexity. Mutual information is a quality metric used to evaluate correspondence for a given transformation [19], and has been extensively applied to medical image registration [20]. However, for infrared-visible images, it might be effective only on a small portion of an image, such as the edges [18] and the foreground [6], since textures in the two sources are significantly different. Therefore, intensity-based methods are not credible for our task.

Feature-based methods involve the extraction of a variety of features for registration, such as points, boundaries and so on. Points are the simplest and most universal feature [8,22], and some point registration methods, such as RPM-VFC [23], have been developed. However, the direct detection of points in infrared-visible images is unreliable due to differences in intensity or texture. Boundaries are commonly captured by both sensors, so the features on boundaries are often considered. In this case, using edges [9,10] or feature points on edges [7,24] is the most popular solution. The curvature scale space [7,24] has been used to extract keypoints on edges. Additionally, [25] aligned the edge maps with a regularized Gaussian fields criterion. Another solution involves using the contours of silhouettes [13,14,26]. The work in [13] directly used shape contours, whereas [14,26] introduced discrete curve evolution (DCE) to extract keypoints on contours. However, boundaries are not identical in infrared-visible images, and these methods might lead to inappropriate features being smuggled in during the calculation of the transformation.

For video pairs with moving objects, motion information is provided in addition to intensity and feature information. Hence, methods based on motion information have been discussed [11,12,27,28]. A method based on optical flow was proposed in [12], but accurately calculating optical flow is difficult and time-consuming. Another method uses trajectories obtained through tracking [11,27,28]. For this method, the centroids or the top points of moving objects (often used as matching points) are strongly influenced by any inaccuracies in the estimated silhouette.

Our proposed method is related to the work in [13,14], which utilized contours of silhouettes to implement registration. We believe that more accurate registration can be achieved by considering the real edges of targets and, hence, build a novel framework based on CSS keypoints [7]. Moreover, we find that the motion information concerning targets can provide a useful condition for keypoint (feature) matching. Therefore, our framework adopts a hierarchical model to combine motion and feature information.

## 3. Registration Framework

**Figure 1:**
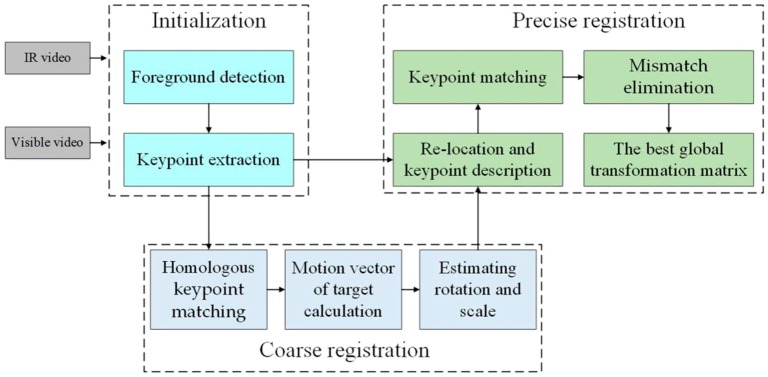
The flowchart of the proposed framework.

Figure 1 shows the flowchart of our framework, which consists of three stages: (1) initialization, (2) coarse registration, and (3) precise registration. During initialization, the foreground is extracted using the method presented in [29]. From the foreground region, we use the CSS algorithm [7] to detect keypoints on the edges of the targets. For a given pair of videos, the keypoints and foregrounds of all images are saved for subsequent processing. In coarse registration, we continuously estimate the motion vectors of the targets based on homologous keypoint matching in selected adjacent frames. With these vectors, scale and rotation are approximately calculated for the pair using the Random Sample Consensus (RANSAC) algorithm [30]. In precise registration, we first re-locate targets and keypoints in the given frames. The keypoints are then matched using NL and HOE descriptors [8,31]. Following this, mismatches are eliminated by counting the matching directions of correspondences. Lastly, we save keypoints from different frames in a reservoir, which are updated based on the matching metric to obtain the best global transformation matrix. Our proposed framework is described thoroughly in subsequent sections, where the left and right images represent infrared and visible images, respectively.

### 3.1. Theory of the Proposed Framework

**Figure 2:**
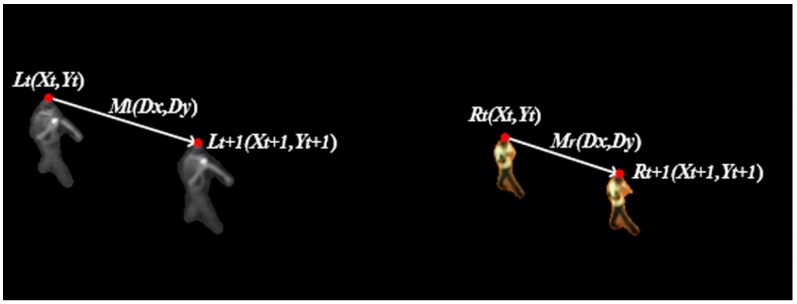
The relationships between the locations of the targets (red dots) and between the motion vectors of the targets (white lines).

The locations of the targets between infrared and visible videos are affected by all parameters in a transformation matrix. However, the motion vectors of the targets are not influenced by translations, regardless of the type of matrix. Suppose that at the moment t, the locations of a target in infrared and visible images are $L_{t}(X_{t}^{l},Y_{t}^{l})$ and $R_{t}(X_{t}^{r},Y_{t}^{r})$, respectively. At t+1, its locations are $L_{t+1}(X_{t+1}^{l},Y_{t+1}^{l})$ and $R_{t+1}(X_{t+1}^{r},Y_{t+1}^{r})$, respectively, as shown in Figure 2, where the location of the target is expressed by its top point.

For an affine matrix with scale S, rotation θ, and translations $T_{x}$ and $T_{y}$, the relationship between the locations of targets can be shown as:

(1) $$\left[\begin{array}{c} Y_{t}^{l}\\ X_{t}^{l} \end{array}\right]=S\left[\begin{array}{cc} \cos\theta & \sin\theta \\ -\sin\theta & \cos\theta \end{array}\right]\left[\begin{array}{c} Y_{t}^{r}\\ X_{t}^{r} \end{array}\right]+\left[\begin{array}{c} Ty\\ Tx \end{array}\right]\;\left[\begin{array}{c} Y_{t+1}^{l}\\ X_{t+1}^{l} \end{array}\right]=S\left[\begin{array}{cc} \cos\theta & \sin\theta \\ -\sin\theta & \cos\theta \end{array}\right]\left[\begin{array}{c} Y_{t+1}^{r}\\ X_{t+1}^{r} \end{array}\right]+\left[\begin{array}{c} Ty\\ Tx \end{array}\right]$$

By obtaining the difference between the target’s positions at different times, the relationship between the motion vectors of targets $M_{l}(Dx^{l},Dy^{l})$ and $M_{r}(Dx^{r},Dy^{r})$ can be described by:

(2) $$\left[\begin{array}{c} Dy^{l}\\ Dx^{l} \end{array}\right]=S\left[\begin{array}{cc} \cos\theta & \sin\theta \\ -\sin\theta & \cos\theta \end{array}\right]\left[\begin{array}{c} Dy^{r}\\ Dx^{r} \end{array}\right]$$

According to Equation (2), we find that once we obtain a pair of motion vectors of the targets, the scale and rotation can be calculated. Inspired by this idea, we build a hierarchical registration framework where we first calculate the motion vectors of the targets, which is crucial to find an accurate global homography. Even though we adopt an affine matrix with four parameters, our framework is applicable to any 2D homography. When using other matrices, we only need to take advantage of different numbers of motion vectors of the targets in the framework.

### 3.2. Initialization

**Figure 3:**
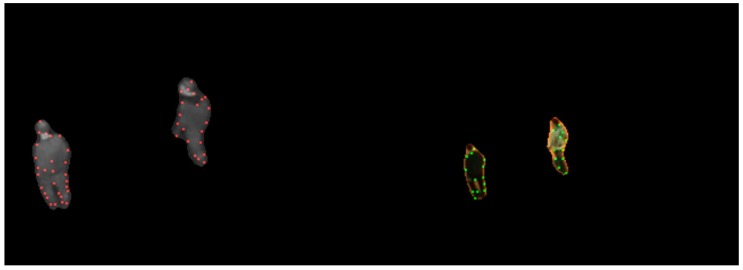
Keypoints found using the CSS corner detection algorithm in infrared (red dots) and in visible images (green dots).

For a given video pair, keypoints need to be extracted from each image. Foreground detection is first performed using the algorithm proposed in [29], which subtracts the background using a statistical background model built using color and binary features, and dynamically updated by feedback mechanisms. Since raw boundaries are not very reliable and using all boundary points is time-consuming, the CSS algorithm [7] that locates keypoints precisely with low computational complexity is then used to extract keypoints from the foregrounds. Being different from the methods used in [13,14], the algorithm extracts keypoints on the Canny edges of actual targets rather than the boundaries of the foregrounds. It can provide more accurate keypoints because it is not influenced by deviations in foreground detection. Figure 3 shows the detected keypoints, where only a part of them are appropriate. This has no effect on our method because we do not directly use keypoint matching. In the initialization, foregrounds and keypoints of the entire video pair are saved.

### 3.3. Coarse Registration

To estimate scale and rotation for the entire video pair, we propose a simple method to calculate the motion vectors of targets. It is based on matching homologous keypoints in adjacent frames, as described below.

#### 3.3.1. Homologous Keypoint Matching

Not all adjacent frames are useful for calculating the motion vectors of targets. Hence, we select appropriate adjacent frames using two conditions: (1) For each image in the infrared and corresponding visible adjacent frames, $N>0.5*N_{max}$ must be tenable, where N is the number of keypoints in the image, and $N_{max}$ is the maximum number of single-frame keypoints in the corresponding video; and (2) The number of infrared and visible targets is always identical in adjacent frames. When these conditions are met, we match homologous keypoints in infrared or corresponding visible adjacent frames.

Homologous images reflect the same phenomena of the observed scene. Thus, we adopt HOG [32] to describe the keypoints to improve the accuracy of matching. Taking a keypoint as the center, we first calculate the gradient orientation ($0^{\circ }-180^{\circ }$) of every pixel in a 16 × 16 pixels block of four 8 × 8 pixels cells. We then count gradient orientations in each cell, and add Gaussian weights to generate nine orientation bins of the histogram. After cascading and normalizing the orientation bins of the four cells, the keypoint is described by a 36-dimensional HOG descriptor. Lastly, we match keypoints in adjacent frames by minimizing the Euclidean distance between descriptors.

**Figure 4:**
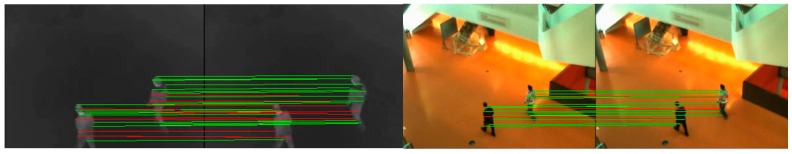
Homologous matched keypoint pairs obtained in infrared images (**the left**) and in visible images (**the right**). The red lines represent mismatches and the green the correct matches.

Figure 4 shows homologous matched keypoint pairs in the infrared and corresponding visible adjacent frames. We found that only a small part of homologous correspondences are unfaithful, and the results are sufficiently reliable to calculate the motion vectors of targets.

#### 3.3.2. Calculating the Motion Vectors of Targets

For the selected adjacent frames, the interframe motion vectors of the targets are obtained based on homologous correspondences. Algorithm 1 shows the steps of calculating the interframe motion vector of the target composed of two loops. In the outer loop, we calculate the motion vector of a correspondence with:

(3) $$\left[\begin{array}{c} dy\\ dx \end{array}\right]=\left[\begin{array}{c} Y_{t+1}\\ X_{t+1} \end{array}\right]-\left[\begin{array}{c} Y_{t}\\ X_{t} \end{array}\right]$$

where $\left(X_{t},Y_{t}\right)$ and $\left(X_{t+1},Y_{t+1}\right)$ are the locations of two keypoints in the correspondence. We then determine the number of inliers in the motion vector, which is dealt with in the inner loop. In this loop, the transformed Euclidean distance error of every correspondence is calculated according to:

(4) $$D=\sqrt{{\left(X_{t+1}-X_{t}-dx\right)}^{2}+{\left(Y_{t+1}-Y_{t}-dy\right)}^{2}}$$

If error D is less than the threshold $D_{th}$ (typically, $D_{th}=2\mathrm{pixels}$), the pair of points is viewed as an inlier of the motion vector. Finally, we select the motion vector with the most inliers as the interframe motion vector of target [Dy,Dx].

**Algorithm 1.** Interframe Motion Vector of Target Calculation**Repeat**
N
**times** (N is the number of homologous correspondences.)1. Pick a homologous correspondence in sequence.2. Calculate the motion vector of the correspondence [dy,dx].3. Estimate the number of inliers in the vector.**Repeat**
N
**times**• Pick a homologous correspondence in sequence.• Calculate the transformed Euclidean distance error D.• If $D<D_{th}$, the correspondence is considered as an inlier.Select the motion vector with the most inliers as the interframe motion vector of target [Dy,Dx].

#### 3.3.3. Scale and Rotation Estimation

For an infrared or visible video, we obtain multiple interframe motion vectors of the targets. To reduce the influence of false motion vectors, the RANSAC algorithm [30] is used to find the scale and rotation. At each iteration of the algorithm, we select K pairs of interframe motion vectors of the targets at random. We then accumulate the selected infrared and visible vectors, respectively, with:

(5) $$\left[Dy_{T}^{L},Dx_{T}^{L}\right]=\sum_{i=1}^{K}\left[Dy_{i}^{L},Dx_{i}^{L}\right]$$

where L∈{IR,Visible}, $\left(Dy_{i}^{L},Dx_{i}^{L}\right)$ is the *i*th interframe motion vector of the target, and $\left(Dy_{T}^{L},Dx_{T}^{L}\right)$ is the total motion vector of the targets. In our experiment, *K* = 20. We calculate rotation and scale using the pair of total motion vectors of the targets according to Equation (2). The Euclidean distance errors between the transformed infrared interframe motion vectors and their corresponding visible vectors are then calculated. When the error of a pair of interframe motion vectors is smaller than the threshold *T* (*T* = 2), this pair is viewed as an inlier. The scale and rotation with the most inliers are the results of coarse registration.

### 3.4. Precise Registration

Since directly matching infrared and visible CSS keypoints yields poor quality, we propose a novel strategy for keypoint matching, as described below.

#### 3.4.1. Re-Location and Keypoint Description

**Figure 5:**
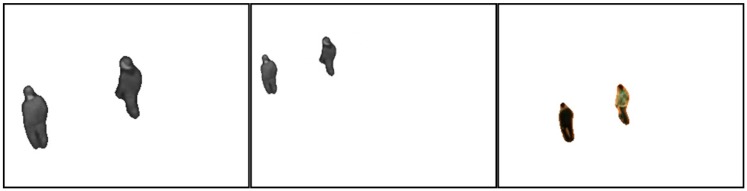
Infrared targets obtained before carrying out transformation (**left**) and after the transformation (**middle**); and the visible target without transformation (**right**).

We re-locate the infrared targets (foregrounds) and keypoints to eliminate the influence of scale and rotation. The targets are first transformed (bilinear interpolation) using the scale and rotation obtained in coarse registration. Figure 5 shows the targets before and after the transformation. The keypoints are then re-located by using:

(6) $$\left[\begin{array}{c} Y^{n}\\ X^{n} \end{array}\right]=\frac{1}{S_{c}}\left[\begin{array}{cc} \cos\theta_{c} & -\sin\theta_{c}\\ \sin\theta_{c} & \cos\theta_{c} \end{array}\right]\left[\begin{array}{c} Y\\ X \end{array}\right]$$

where [Y,X] and $\left[Y^{n},X^{n}\right]$ are the locations of a keypoint before and after re-location, respectively, and $S_{c}$ and $\theta_{c}$ are the results of coarse registration. Following this, the influence of scale and rotation on the keypoints and targets can be ignored. Therefore, we can use two descriptors for a keypoint, as follows:

- $P\left(Y_{N},X_{N}\right)$: Its normalized location (NL). It is calculated by:
  (7) $$\left[Y_{N},X_{N}\right]=\left[Y,X\right]-\left[Y_{c},X_{c}\right]$$
  where $\left[Y_{c},X_{c}\right]$ is the centroid of the foreground, and [Y,X] is the position of the keypoint. When foreground detection and re-location are both perfect, the NL descriptors of a correct correspondence are identical.
- E: Its histogram of edge orientation (HOE, [8,31,32]). Its construction is similar to that of the HOG (in Section 3.3.1). However, HOE only considers the orientations of the Canny edges of the targets, whereas HOG uses the gradient orientation of each pixel. It abandons the information in low-relevance regions, and uses the similarity between infrared and visible edges. The HOE descriptor is represented by:
  (8) $$E_{i}=P\left(i\right),i=1,2,\ldots ,36$$
  where i is an index of the histogram, and P(i) is the proportion of points with index i.

#### 3.4.2. Matching

Having described all infrared and visible keypoints, we need to define some metrics for the matching process:

- $D_{p}$: The normalized Euclidean distance between two keypoints:
  (9) $$D_{p}=\left|P^{l}-P^{r}\right|$$
  where $P^{l}$ and $P^{r}$ are the normalized locations of an infrared and a visible keypoint, respectively.
- $D_{E}$: The difference between the HOE descriptors of two keypoints:
  (10) $$D_{E}=\sum_{i=1}^{36}\left|E_{i}^{l}-E_{i}^{r}\right|$$
  where $E_{i}^{l}$ and $E_{i}^{r}$ are the *i*th component of an infrared and a visible HOE descriptor, respectively.

During the matching process, we first consider the normalized Euclidean distance $D_{p}$ between a pair of keypoints from the given infrared and visible images. If $D_{p}<D_{th}$, the keypoint pair is a possible match; otherwise, we ignore it and judge another pair. In this step, all possible pairs are considered. We temporarily save all possible matches because there may be more than one matching point in the visible image for some infrared points. Lastly, if there is only one possible match for an infrared keypoint, we view this as the best match. Otherwise, we select the best match by minimizing $D_{E}$. Considering the errors of foreground detection and re-location, we used $D_{th}=10$ pixels.

#### 3.4.3. Mismatch Elimination

There are a few mismatches in the matched keypoint pairs. For instance, some infrared keypoints are matched to the same one keypoint in the visible image. This situation may occur due to the lack of an obvious difference between an infrared or a visible HOE descriptor and others. Therefore, we need a mechanism to remove mismatches.

We define the matching direction of a match as:

(11) $$\theta^{R}=\frac{Y^{l}-Y^{r}}{X^{l}-X^{r}+\varepsilon }$$

where $\left(Y^{l},X^{l}\right)$ and $\left(Y^{r},X^{r}\right)$ are the locations of an infrared and a visible keypoint in the match, respectively, and ε is a balancing factor set to the width of the infrared image. Following re-location, we can assume that the locations of the keypoints are only affected by translations; hence, the matching direction of a correct match is close to a fixed value (the fixed value is Ty/(Tx+ε), which is easily derived from Equation (1) by ignoring the scale and rotation). On the contrary, the matching direction of a fault match is uncertain. Under such a circumstance, we propose an algorithm to eliminate mismatches based on the matching directions of matched keypoint pairs.

As introduced in Algorithm 2, we first calculate the matching directions of all matches according to Equation (11). Then, the code of every match is determined by:

(12) $$C_{i}=floor((\theta_{i}^{R}-\theta_{\min}^{R})/\theta_{wid}^{R})+1$$

where $\theta_{i}^{R}$ is the matching direction of the $i^{th}$ match, $\theta_{\min}^{R}$ is the minimum of all matching directions, and $\theta_{wid}^{R}$ is coding width. Experiments showed that we obtain the best results when $\theta_{wid}^{R}$ = 0.01. Finally, we calculate the histogram of matching direction. When the ratio of its maximum to secondary maximum is more than α (typically, α=1.2), we save the matches with code corresponding to the maximum.
**Algorithm 2.** Mismatch Elimination Based on Matching Direction1. Calculate the matching direction of each match.2. Encode every match using its matching direction.3. For every code, count the number of matches with this code to create the histogram of matching direction.4. Find the maximum value of histogram $M_{1}$ and the secondary maximum value $M_{2}$.5. If $M_{1}>\alpha \cdot M_{2}$, save the matches with code of the maximum; otherwise, abandon all.

**Figure 6:**
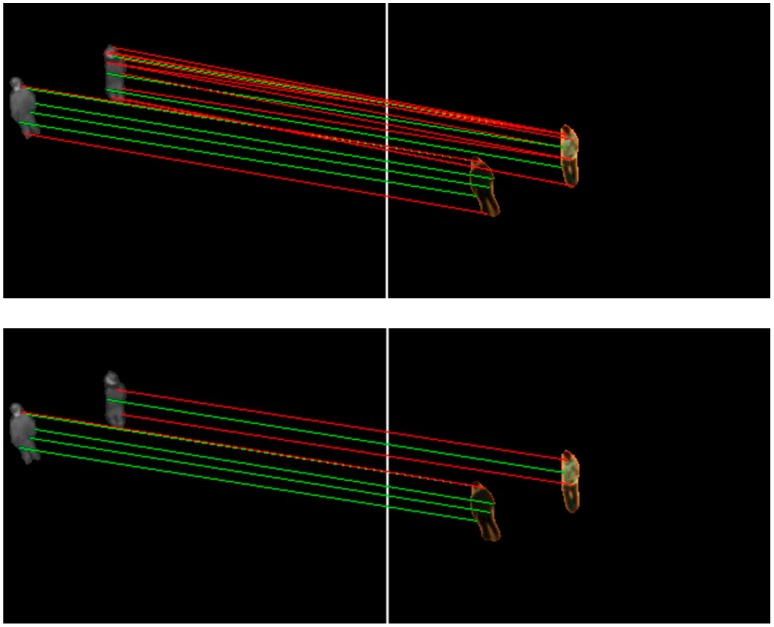
Matched keypoint pairs obtained before using the elimination of mismatches (the upper) and after using the elimination (the lower). The red lines represent mismatches and the green the correct matches.

Figure 6 shows matches before and after the elimination of mismatches. We found that our algorithm could eliminate most mismatches.

#### 3.4.4. Finding the Best Global Homography

If we only use matches from a single frame pair for registration, it is not possible to find an accurate global homography, especially when the observed scene is large and does not follow the planar ground assumption. To solve this problem, we can save matches from different frames in a reservoir. However, previously-proposed reservoirs have some certain disadvantages. In [14], a FIFO (first-in, first-out) reservoir was used to retain matches from 100 frames. However, when the movement of the targets of interest is not obvious during 100 frames, or foreground detection is continuously noisy, it cannot save a sufficient number of typical matches to calculate the homography. The authors of [13] used a reservoir where matches identified as persistent outliers based on the RANSAC algorithm are randomly replaced. However, the RANSAC algorithm is unstable, and may produce false homography in consecutive frames, particularly at the beginning of registration. At this time, it cannot accurately distinguish outliers. Hence, we found a new reservoir, in which we replace matches based on the HOE matching metric.

For a given reservoir $R=\left\{p_{1},p_{2},\ldots ,p_{N}\right\}$ containing N previously-found matches, we record the HOE matching metric of each point pair $V=\left\{v_{1},v_{2},\ldots ,v_{N}\right\}$, which is calculated by Equation (10). In the reservoir, matches with HOE matching metrics greater than the median are regarded as outliers. When a new match is found, we pick one of the outliers in the reservoir at random and replace it. In practice, our reservoir is never saturated, and new matches are always swapped in. With all of the matches in, we calculate the given global homography using the standard RANSAC algorithm [30].

Our scene of interest does not always comply with the planar ground assumption; thus, the goal of our method is to find a best global homography to ensure accuracy of registration, not only for the targets of interest in the given frame, but also those in the entire video pair, even if non-planar registration is involved. To achieve this goal, we use the given global homography to update the best global homography according to the method of homography smoothing described in [13]. The total best global homography is lastly found by combining the results of coarse and precise registration:

(13) $$\theta_{t}=\theta_{c}+\theta_{p},\;S_{t}=S_{c}\times S_{p}\;Ty_{t}=S_{c}\times (Ty_{p}\times \cos(\theta_{c})+Tx_{p}\times \sin(\theta_{c})),\;Tx_{t}=S_{c}\times (Tx_{p}\times \cos(\theta_{c})-Ty_{p}\times \sin(\theta_{c}))$$

where $S_{c}$ and $\theta_{c}$ are the rotation and scale obtained in coarse registration, respectively, and $S_{p}$, $\theta_{p}$, $Ty_{p}$ and $Tx_{p}$ are the scale, rotation, and the translations obtained in precise registration.

## 4. Experiment and Analysis

### 4.1. Experiment

In this section, we describe tests on the proposed framework using several nearly planar scenes. The LITIV dataset provided by Torabi [11] was employed in our experiment. It is a publicly available dataset for infrared-visible registration containing nine video pairs of resolution 240 × 320 and lengths varying between 200 and 1200 frames. Although these were taken with different sensor baselines at different orientations, all scenes are almost planar because the targets were always viewed from afar. Furthermore, it provides ground-truth transformation matrices found by manually selecting corresponding point pairs. These were used to produce the results of the ground truth global registration.

We compared our framework with two state-of-the-art global registration methods, both of which are based on keypoint matching. The first one directly uses shape contours and a reservoir based on a voting scheme (Charles et al. [13]), and the second uses DCE keypoints and a FIFO reservoir (Sonn et al. [14]). For fairness, we used the same foreground detection results for all methods. All methods were evaluated by the same error metric, and adopted a single parameter set for all video pairs.

The parameters used by all methods mainly contain $T_{S}$, $C_{\min}$ and $C_{\max}$. $T_{S}$ indicates the Euclidean distance error used in the RANSAC algorithm to calculate the given global homography. Typically, $T_{s}=5$. $C_{\min}$ indicates a threshold for matches in a reservoir. Once the number of matches in the reservoir is greater than the threshold, the RANSAC algorithm starts to calculate the given global homography. In our experiment, the calculation should be started promptly when the matches are sufficient. Therefore, it is set to 5. $C_{\max}$ expresses the maximum number of matches that can be saved in the same reservoir. For the proposed method and the one in [13], $C_{\max}=100$. The reservoir used in [14] can save matches from 100 consecutive frames. Generally speaking, $C_{\max}$ has a significant influence on the registration accuracy. With more matches in a reservoir, higher accuracy can be achieved. However, computation time is likewise longer with a larger reservoir.

To quantify the performance of these methods, we first needed the binary polygons sets. The construction method was proposed in [11]: for an infrared-visible video pair, they first manually selected some identifiable and matchable points in the infrared image, and manually found the corresponding points in the visible image. They then connected them to create binary polygons. In our case, we used the binary polygons provided by [13], and then evaluated each method with overlap error:

(14) $$OE=1-\frac{P_{l}\cap P_{r}}{P_{l}\cup P_{r}}$$

where $P_{l}$ and $P_{r}$ are the transformed infrared polygon and the visible polygon, respectively. In practice, the overlap error between the transformed infrared foreground and the visible foreground has been used in homography smoothing [13]. It was adopted to find the best global homography in our framework. By using binary polygons instead of foregrounds to calculate the overlap error, we eliminated the influence of inaccurate foreground detection.

### 4.2. Analysis

**Figure 7:**
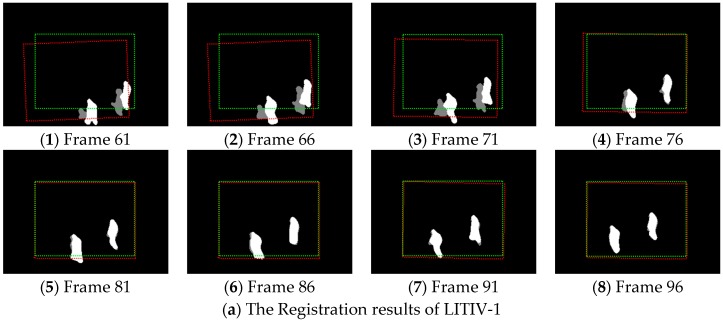

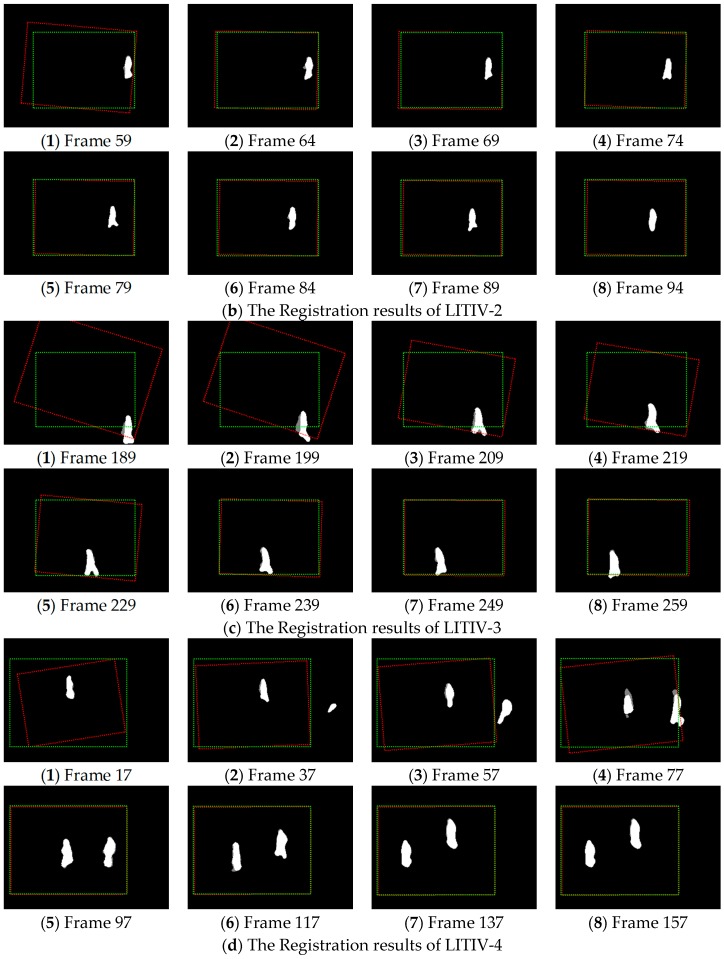
(**a**–**d**) Registration results obtained at various moments of the first, second, third and fourth video pairs of the LITIV dataset using our proposed framework. The red-dashed rectangle shows the estimated transformation applied to the infrared image boundary, and the green one shows the ground-truth transformation applied to the same boundary.

How our framework performs in the first four video pairs of the studied dataset is shown in Figure 7.

We can see that a transformation is found soon after a target first becomes visible in both the infrared and the visible videos (this happens at different moments in each video pair; the earliest results are shown in Figure 7 (1) of each result sequence), even if these videos were taken at various sensor positions. At this time, the alignment of foregrounds is acceptable, except LITIV-1. In LITIV-1, an infrared target is seriously incomplete when we first calculate the homography. Hence, we cannot obtain correct matches in precise registration, which results in the stagger of the matched foregrounds. However, the homography is refined to register the foregrounds sufficiently well over time in all pairs. In LITIV-1 or LITIV-2, an accurate transformation is found less than 30 frames after first calculating the transformation, although the movement of the targets is faint.

**Figure 8:**
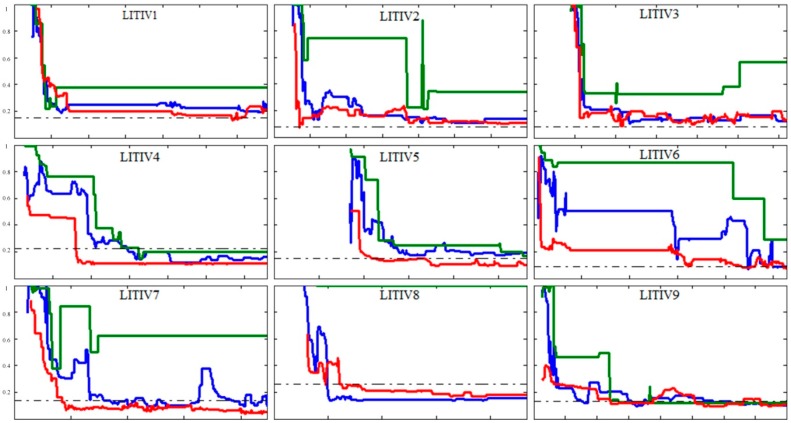
Polygon overlap errors obtained using our framework (solid red), the method of [13] (solid blue), the method of [14] (solid green), and the ground-truth homography (dot-dashed gray) for the full lengths of all sequence pairs of the LITIV dataset.

In our experiments, the overlap error was used to assess our method. To reflect the global performance of our method, we drew error-to-time curves for our method and compared them with those of the other two methods [13,14], as shown in Figure 8.

We can find that our method reaches lower overlap errors at faster convergence speeds than [14], and stabilizes at those levels over longer times for all video pairs. Moreover, it has no trouble estimating the homography for LITIV-8, unlike [14], which is unable to find a useful homography for the pair.

**Figure 9:**
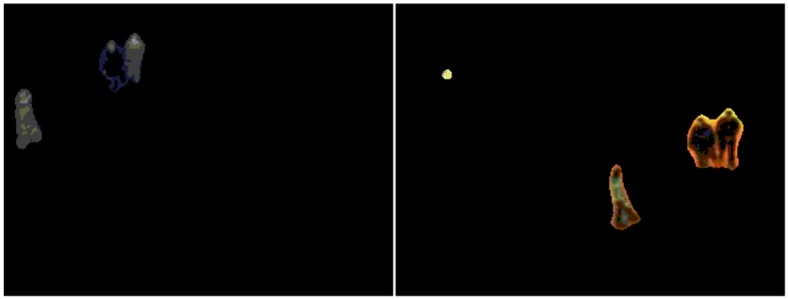
Foregrounds extracted from infrared (**the left**) and from visible (**the right**) images in Frame 366 of LITIV-8.

Then, we discuss the comparison results between our method and [13]. For LITIV-2, LITIV-3, and LITIV-9, the errors of our method are close to those incurred by [13] in most images, but our method reaches lower errors at faster convergence speeds; For LITIV-1, LITIV-4, LITIV-5, and LITIV-7, our method reaches significantly lower errors and stabilizes at these levels more often; For LITIV-6, [13] reaches a smaller minimum overlap error. However, the integrated error level of our method is lower. Hence, the proposed method outperforms [13] for these video pairs. Being different from these results, the errors of our method are higher than those of [13] for most images in LITIV-8. Which is mainly because of two factors: first, foreground detection is poor in some frames, as shown in Figure 9, and second, re-location cannot adequately remove the influence of scale and rotation on the whole scene, since the non-planar characteristic of the scene is obvious. These factors might produce some inaccurate matches in precise registration. The results of [13] reflects better performance for this pair because this method does not need to re-locate keypoints, and does not deal with the distances between keypoint pairs. However, the disadvantages of the proposed method are not serious (the matches are almost accurate), so we obtain acceptable results close to [13].

Our method succeeds in excelling ground truth homography in all but LITIV-2 and LITIV-3. It is possible and desirable because the ground truth is not perfect. It was found manually because there is a margin of error. Furthermore, the ground truth provides an ideal transformation only for a planar scene, but the binary polygons found on the targets of interest do not fully follow the planar ground assumption. Hence, methods that aim to register these targets can obtain lower errors.

As shown in Table 1, our method reaches smaller minimum overlap errors than [13] for all pairs. Further, the minimum errors are less than half of those of [14] in the second, third, sixth, seventh, and eighth pairs. Our method also reaches lower minimum errors than [13] for all pairs, except LITIV-6 and LITIV-8, where the differences between the two are small. The table also shows that our method reaches lower errors than the ground truth for all but LITIV-2 and LITIV-3.

We adopted the average overlap errors to summarize the results, which can intuitively represent the overall capacity of a method. As shown in Table 2, our method is better than [13,14] for all but LITIV-8. This is because our method has three features: (1) the keypoints used in our method are more accurate. We extracted keypoints on the Canny edges of real targets, which are not influenced by errors in foreground detection (in [13,14] keypoints were extracted on the contours of foregrounds, which are easily influenced); (2) we built a stricter registration framework by combining feature and motion information. Based on coarse registration, we used novel descriptors and the mechanism of eliminating mismatches to improve the accuracy of keypoint matching; and (3) we used a reservoir based on the HOE matching metric, which can save more typical matches than those used in [13,14]. Therefore, our method outperforms these two methods.

When operating directly on the foregrounds provided by the target detection algorithm [29], the average computing time for one frame in each sequence is shown in Table 3. For each sequence pair, we can find that the average speed of our proposed method varies between 6 and 18 frames per second. Actually, the speed is dependent on the number of targets in the scene (we finished our experiment using MATLAB R2013b, on an Intel(R) Core(TM) i5-4590, 3.30 GHz CPU,4 GB RAM, Win7 x64 system, in Beijing, China).

## 5. Conclusions

In this paper, we presented a hierarchical framework that combines motion and feature information relying on CSS keypoints to register thermal-visible videos. In the framework, we used the motion vectors of the targets to approximate scale and rotation in coarse registration step. Based on the results, a strict strategy of keypoint matching was proposed to accurately calculate the global transformation matrix in a precise registration step. A reservoir updated based on the difference of HOE also allowed our method yield better results. The results of an experiment showed that our method outperformed two recent state-of-the-art global registration methods in almost all tested video pairs. Moreover, it managed to manually align binary polygon sets based on scene structures, the results of which were preferable to the ground truth homography in a majority of sequences.

## Figures and Tables

**Table 1:** Minimum overlap errors for all video sequence pairs of the LITIV dataset (bold entries indicate the best results).

Sequence Pair	Proposed	Charles et al. [13]	Sonn et al. [14]	Ground-Truth
LITIV-1	**0.135**	0.187	0.217	0.149
LITIV-2	**0.083**	0.106	0.214	0.078
LITIV-3	**0.101**	0.108	0.258	0.080
LITIV-4	**0.109**	0.118	0.152	0.221
LITIV-5	**0.102**	0.172	0.167	0.150
LITIV-6	0.083	**0.069**	0.289	0.088
LITIV-7	**0.052**	0.091	0.379	0.136
LITIV-8	0.176	**0.137**	1.000	0.260
LITIV-9	**0.093**	0.095	0.117	0.134

**Table 2:** Average overlap errors for all video sequence pairs of the LITIV dataset (bold entries indicate the best results).

Sequence Pair	Proposed	Charles et al. [13]	Sonn et al. [14]	Ground-Truth
LITIV-1	**0.226**	0.266	0.399	0.149
LITIV-2	**0.162**	0.205	0.538	0.078
LITIV-3	**0.187**	0.193	0.423	0.080
LITIV-4	**0.198**	0.312	0.399	0.221
LITIV-5	**0.151**	0.267	0.339	0.150
LITIV-6	**0.190**	0.413	0.785	0.088
LITIV-7	**0.136**	0.257	0.668	0.136
LITIV-8	0.237	**0.204**	1.000	0.260
LITIV-9	**0.173**	0.185	0.241	0.134

**Table 3:** Average computing time for one frame.

Sequence Pair.	LITIV-1	LITIV-2	LITIV-3	LITIV-4	LITIV-5	LITIV-6	LITIV-7	LITIV-8	LITIV-9
Computing times (s)	0.056	0.142	0.085	0.163	0.110	0.116	0.108	0.103	0.101
